# The Complex Toxicity of Tetracycline with Polystyrene Spheres on Gastric Cancer Cells

**DOI:** 10.3390/ijerph17082808

**Published:** 2020-04-19

**Authors:** Xiemin Yan, Yuanyuan Zhang, Yuqin Lu, Lei He, Junhao Qu, Chunxia Zhou, Pengzhi Hong, Shengli Sun, Hui Zhao, Yanqiu Liang, Lei Ren, Yueqin Zhang, Jinjun Chen, Chengyong Li

**Affiliations:** 1 College of Food Science and Technology, Guangdong Ocean University, Zhanjiang 524088, China; xiemin_y@163.com (X.Y.); 15975997422@163.com (J.Q.); zhoucx@gdou.edu.cn (C.Z.); hongpz@gdou.edu.cn (P.H.); 2 Shenzhen Institute of Guangdong Ocean University, Shenzhen 518108, China; zyyla92@126.com; 3 School of Chemistry and Environment, Guangdong Ocean University, Zhanjiang 524088, China; rebeccaaax@163.com (Y.L.); sunsl@gdou.edu.cn (S.S.); huizhao1978@163.com (H.Z.); liangyanqiu11@126.com (Y.L.); 4 Southern Marine Science and Engineering Guangdong Laboratory (Zhanjiang), Zhanjiang 524088, China; 5 College of Agriculture, Guangdong Ocean University, Zhanjiang 524088, China; renlei@gdou.edu.cn (L.R.); yqzhang1982@163.com (Y.Z.); chenjj@gdou.edu.cn (J.C.)

**Keywords:** microplastics, nanoplastics, adsorption, tetracycline, AGS cells

## Abstract

Nowadays, microplastics (MPs) exist widely in the marine. The surface has strong adsorption capacity for antibiotics in natural environments, and the cytotoxicity of complex are poorly understood. In the study, 500 nm polystyrene (PS-MPs) and 60 nm nanoplastics (PS-NPs) were synthesized. The adsorption of PS to tetracycline (TC) was studied and their toxicity to gastric cancer cells (AGS) was researched. The adsorption experimental results show that PS absorbing capacity increased with increasing TC concentrations. The defense mechanism results show that 60 nm PS-NPs, 500 nm PS-MPs and their complex induce different damage to AGS cells. Furthermore, 600 mg/L PS-NPs and PS-MPs decline cell viability, induce oxidation stress and cause apoptosis. There is more serious damage of 60 nm PS-NPs than 500 nm PS-MPs in cell viability and intracellular reactive oxygen species (ROS). DNA are also damaged by 60 nm PS-NPs and PS-TC NPs, 500 nm PS-MPs and PS-TC MPs, and 60 nm PS-NPs damage DNA more serious than 500 nm PS-MPs. Moreover, 60 nm PS-NPs and PS-TC NPs seem to promote bcl-2 associated X protein (Bax) overexpression. All treatments provided us with evidence on how PS-NPs, PS-MPs and their compounds damaged AGS cells.

## 1. Introduction

For many years, plastics have been applied in numerous fields, such as packing manufacture, cosmetics, clothing, and so on [1,2]. It is estimated that around 1.15 to 2.41 million tons of plastic waste were dumped into oceans annually [3]. Plastics have been found in hundreds of marine organism species [4,5,6]. Through Ultraviolet (UV) ray exposure and mechanical wear in the environment, plastics break down into small fragments. Particle diameters smaller 5 mm are defined as microplastics (MPs) [7,8,9]. MPs originate from two sources, including primary MPs and secondary MPs. Primary MPs are used as resin pellets in industry or as an ingredient in peelings and shower gels [10]. Secondary MPs are formed from larger plastic items degrading under favorable conditions. MPs smaller than 100 nm are defined as nanoplastics (NPs) [11]. In recent years, MPs have been deemed to be a new environmental pollutant through further investigation. Many researchers have found that a variety of aquatic species ingested MPs, including fish, oyster, and shrimp [12,13,14]. MPs damage their gastrointestinal tract and growth speed and enter their bodies [15,16,17]. The impact of MPs and NPs occur via many ways: their small size facilitates internalization by biota or pathogenic bacteria; and pollutants can absorb on to an MPs’ surface [18,19]. Most significantly, MPs can enter the human body for resultant accumulation through the food chain [20,21,22]. Up to now, there are no obvious data to identify how MPs are hazardous to human health, but the alarm has been sounded.

Nowadays, antibiotics have attracted wide attention as a type of contaminant. Normally, antibiotics are rich in offshore breeding areas, which can enter the offshore environment through sewage discharge [23,24]. Moreover, antibiotics are applied in fisheries extensively, they also exacerbate residue in offshore water [25]. Tetracycline (TC), one of these antibiotics, is widely used to inhibit bacteria growth and kill pathogens. MPs have an extremely strong adsorption capacity on persistent organic pollutants [26,27,28]. What’s more, MPs surface characteristics can be changed in the effect of physical and chemical function in the natural environment [29]. The adsorption capacity of MPs can be enhanced once an amino-group and carboxyl has formed on an MPs surface [30]. Some research found that the main interaction of polystyrene spheres (PS) and TC is electrostatic attraction, and as the existence of benzene, π-π conjugation, polar interactions, and p-p interaction are also present [23,31]. In addition to individual pollution effects, their complex toxicity deserve more attention.

To study the toxicity of PS-MPs, PS-NPs and their combination with TC, gastric cancer cells (AGS) are chosen because they can be in contact with stomach cells first after consumption. In order to study whether nanoplastics can induce a greater negative effect to AGS cells than microplastics, both 500 nm PS-MPs and 60 nm PS-NPs were chosen according to the definition of microplastics and nanoplastics [32]. The toxicity of PS and PS compound TC (PS-TC) to AGS are determined by the basic experiments of cell viability, intracellular reactive oxygen species (ROS) and apoptosis. Comet assay aims to detect DNA damage. The aim is to provide a proof of how PS-MPs, PS-NPs and their compounds damage AGS cells. The study may provide a theoretical support for the negative effects of MPs on human health.

## 2. Materials and Methods

### 2.1. Materials

Styrene monomer and AIBN (2,2′-Azobis (2-methylpropionamide) dihydrochloride) were purchased from Shanghai Maclean Biochemical Technology Co., Ltd. (Shanghai, China). A three-necked bottle, rubber tube, and condensing return pipe were obtained in Zhanjiang Haibo Biotechnology Co., Ltd. (Zhanjiang, China). AGS cell line was acquired from Shanghai Institute of Biosciences Cell Resource Center, Chinese Academy of Sciences. Fetal bovine serum (FBS), trypsin, penicillin/streptomycin were supported by Guangzhou Zuoke Biotechnology Co., Ltd. (Guangzhou, China). The anti-Bax antibody was obtain from Santa Cruz Technology (Santa Cruz, CA, USA). And secondary antibodies used for western blot were purchased from Cell Signaling Technology (Danvers, MA, USA).

### 2.2. Methods

#### 2.2.1. Synthesis of PS-MPs and PS-NPs

PS-MPs and PS-NPs were synthesized through monomer polymerization according to a previous described method [33,34]. Briefly, 500 nm (or 60 nm) PS particles were synthesized in the following process: 90 mL (or 198 mL) deionized water was added into three-necked bottle and warmed up to 55 °C (or 90 °C). After the reaction in the system, oxygen was taken away by nitrogen and 2,2′-Azobisisobutyronitrile (AIBN), as an initiator, was added into the system. The reaction lasted 10 h under nitrogen atmosphere and was cooled with 4 °C circulating water. After the reaction ended, the three-necked bottle was cooled with ice-cold water to room temperature. 500 nm PS-MPs was cleaned using absolute ethanol through 0.22 μm microporous membrane. As for 60 nm PS-NPs, all solutions were loaded into a 30 kDa dialysis bag, and the bags were put into deionized water for 5 days, and deionized water was changed every day. Finally, all samples were dried at 50 °C.

#### 2.2.2. Adsorption Experiments

According to the study by Hans et al. [35], TC has a ultraviolet spectrophotometer (UV-V) response signal. The UV-V absorption peak of TC was determined at 341 nm. The linear range was ascertained. The sorption capacity of TC on PS-NPs and PS-MPs were researched by batch experiments. TC concentrations were 1, 5, 10, 50, and 100 mg/L in the study. Next, 1 g/L PS-NPs and PS-MPs were uniformly dispersed to various TC concentration solutions by ultrasound, respectively. All samples were mixed uniformly in 300 rpm with magnetic stirring. Samples were collected after removing PS-NPs or PS-MPs every hour and 500 nm PS-MPs samples were obtained by filtering 0.22 μm microporous membrane. For 60 nm PS-NPs samples, solutions were acquired through 0.02 μm aluminum oxide membrane to remove PS-NPs. The absorbance values of all samples (1, 2, 3, 4, 5, 6 h) were measured using UV-V (U-3900H, HITACHI) at 341 nm.

#### 2.2.3. Cell Viability Assay

AGS cells cultured in F-12K medium supplemented with 10% FBS, 1% penicillin/streptomycin solution, and 5% CO_2_ at 37 °C incubator. A 200 μL AGS cell suspension (1 × 10^5^–5 × 10^5^ cells/mL) was spread into 48-well plates overnight. Cell viability was measured using an MTS assay kit (Nan Jing Jian Cheng Biotechnology Co., Ltd. Nanjing, China) after exposure to PS-NPs and PS-MPs for 24 h. Absorbance of the mixture was measured at 570 nm using a microplate reader (Synergy H1, BioTek, VT, USA). Cell viability was calculated by comparing with the absorbance of the control group.

#### 2.2.4. Intracellular ROS Analysis

AGS intracellular ROS levels induced by PS-NPs, PS-MPs (50, 100, 200, 400, 600 mg/L), and TC (0.5, 1, 5, 10, 20 mg/L) exposure were detected by a ROS assay kit (DCFH-DA, Beyotime Biotechnology, Shanghai, China). Next, 10 mM DCFH-DA was added to each well of 96-well plate after exposure to PS-NPs, PS-MPs, and TC for 24 h. After incubating under 37 °C for 30 min, AGS cells were washed with PBS. The fluorescence of DCF was detected in the excitation/emission wavelengths of 488/525 nm using an Olympus fluorescence microscope. The normalized DCF fluorescence values of exposed groups were calculated by comparing them with the control group using Image J.

#### 2.2.5. Apoptosis Experiment

AGS cells (2 × 10^5^–5 × 10^5^ cells/mL) were exposed to PS-NPs, PS-MPs, PS-TC NPs, MPs (50, 100, 200, 400, 600 mg/L), and TC (0.5, 1, 5, 10, 20 mg/L) for 24 h. Then all cells were stained with Annexin V-FITC/PI apoptosis kit (Beyotime Biotechnology, Shanghai, China). According to the manufacturer’s instructions, all groups were pictured by an Olympus fluorescence microscope.

#### 2.2.6. Comet Experiment

All treatment cells were collected using trypsinization, and cell suspension concentration was adjusted to 1 × 10^5^ cells/mL with PBS. The slides were coated with 0.5% normal-melting-point agarose as a first layer. After solidification, 0.5% low-melting-point agarose (80 μL) and cell suspension (1 × 10^5^ cells/mL, 20 μL) was poured and mixed on the first layer. Once the agarose coagulated, all samples were put in lysis solution for 2 h. Next, they were transferred to alkaline electrophoresis solution (AES) for 30 min. After the DNA double helical structure was unwound, the electrophoresis was performed at 25 V for 30 min in AES buffer and then neutralized with Tris-HCl buffer (0.1 mM, pH = 13). All bands were dehydrated using absolute ethanol and dried, stained bands were gathered and analyzed.

#### 2.2.7. Analysis of Apoptotic Protein by Western Blot

In order to further research PS-MPs and PS-NPs toxicity, Bax as an apoptotic protein was detected. Six hundred milligrams per liter PS-MPs and PS-NPs co-cultured with AGS cells for 24 h. Next, AGS cells in 6-well plate were washed using PBS, then 60 μL cell lysates with PMSF (1 mM) was added lasted for 30 min on ice. Total proteins were quantified using a BCA protein assay kit (Beyotime Biotechnology, Shanghai, China) and 20 μg protein was separated electrophoretically using a 10% sodium dodecyl sulfate-polyacrylamide gel electrophoresis (SDS-PAGE). All bands were transferred to polyvinylidene fluoride (PVDF) using a wit transfer method. Bands were blocked with 5% skim milk for 2 h, and incubated with primary antibodies at 4 °C overnight. The next day, a secondary antibody was incubated for 2 h at room temperature. All bands were visualized with an enhanced chemiluminescence (ECL) kit (Beyotime Biotechnology, Shanghai, China).

## 3. Results

### 3.1. The Principle of the Study

**Figure 1:**
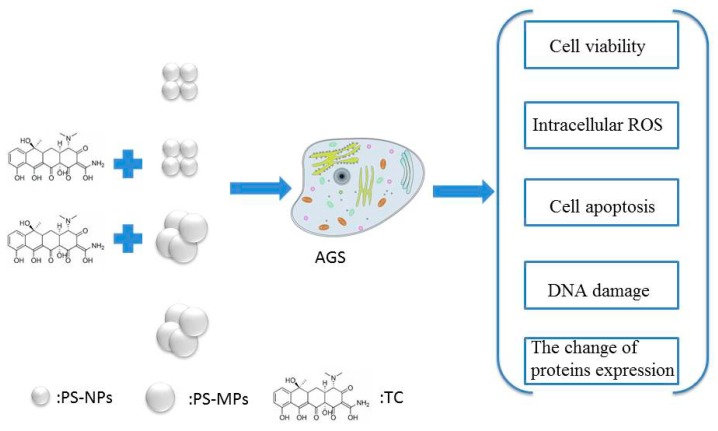
The experimental schematic. Abbreviations including Tetracycline (TC), polystyrene microplastics (PS-MPs) and polystyrene nanoplastics (PS-NPs).

The mechanism is shown in Figure 1. Sixty nanometers PS-NPs and 500 nm PS-MPs were synthesized by monomer polymerization. The bath sorption experiment was treated as following: 1 g/L PS-NPs and PS-MPs are mixed with a different concentration of TC (1, 5, 10, 50, 100 mg/L), respectively. PS-MPs and PS-NPs are removed through a filter every hour. The concentration of TC remaining in solution can be determined according to a liner curve. PS-MPs and PS-NPs co-culture with AGS cells. Cell viability, oxidative stress, and apoptosis as the indicators of cell damage are detected. Comet experiment results confirmed the toxicity in the level of DNA.

### 3.2. The Characteristic of PS Particles

**Figure 2:**
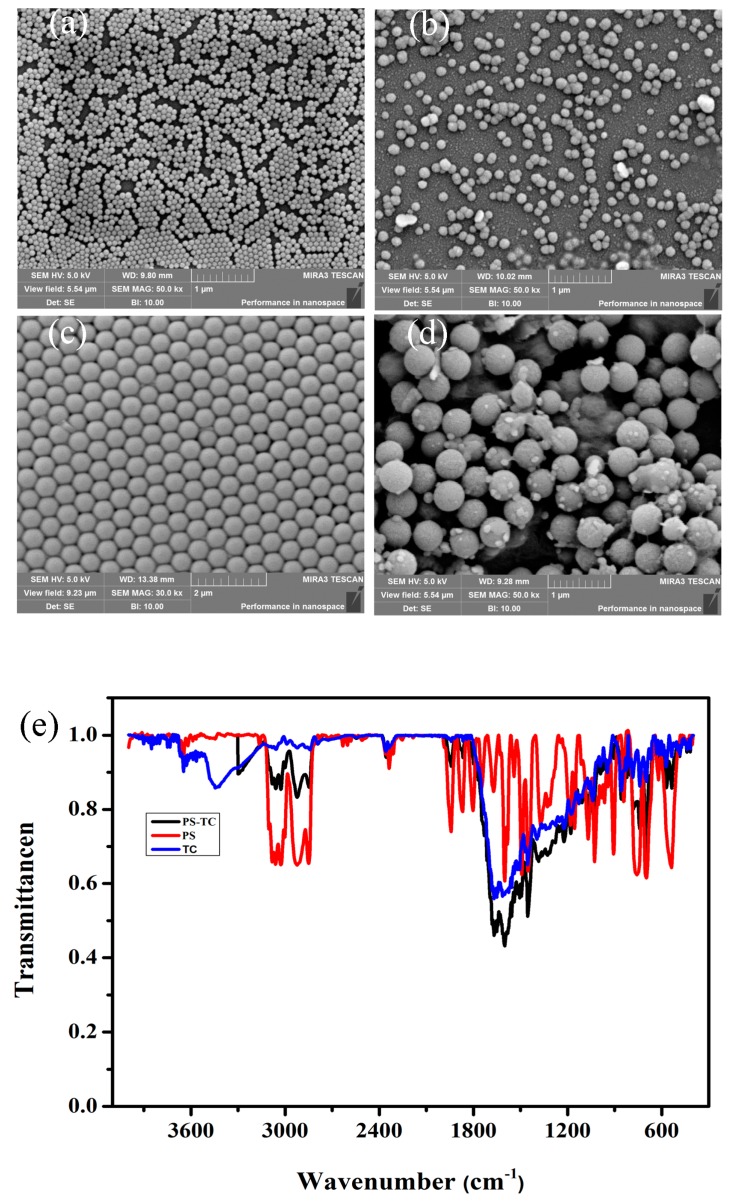
The characteristics of polystyrene spheres (PS). (**a**) 60 nm PS-NPs; (**b**) 60 nm PS-NPs in medium; (**c**) 500 nm PS-MPs; (**d**) 500 nm PS-MPs in medium; (**e**) Fourier transform infrared spectroscopy of PS and PS compound TC (PS-TC).

In order to confirm PS-NPs and PS-MPs were obtained according to the previous research method [34], the sizes of PS-NPs and PS-MPs as depicted in Figure 2a,c were evaluated by SEM. Considering the interaction between PS and medium, the sizes of PS in medium are also measured as described in Figure 2b,d. The results suggest that both 60 nm PS-NPs and 500 nm PS-MPs surface adsorb some proteins. What is more, previous study has identified that MPs’ surface formed corona in medium [36]. Besides, the compound of PS-TC is identified with Fourier transform infrared spectroscopy (FTIR) in Figure 2e. The hydration radius of PS-NPs and PS-MPs are determined at Table 1. The results identify PS particles in medium that are larger than in deionized water. PS sizes become larger after exposure to medium for 24 h because their surface attaches some proteins or protein fragments.

### 3.3. Adsorption Experiment

**Figure 3:**
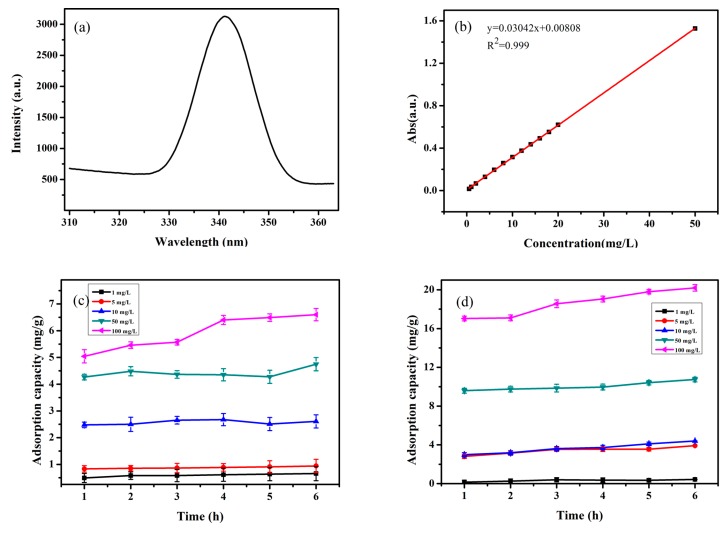
Adsorption experiments. (**a**) The UV adsorption of TC; (**b**) The linear range of TC; (**c**) The adsorption capacity of 500 nm PS-MPs to TC (1, 5, 10, 50, 100 mg/L); (**d**) The adsorption capacity of 60 nm PS-NPs to TC (1, 5, 10, 50, 100 mg/L).

In Figure 3a, it is clear that TC has strong UV-vis adsorption at 341 nm. There is a good linearity ranging from 0.5–50 mg/L in Figure 3b. As shown in Figure 3c,d, with TC concentrations increasing, both 500 nm PS-MPs and 60 nm PS-NPs have an increasing adsorption capacity on TC. A 500 nm PS-MP adsorption quantity is 0.66, 0.94, 2.61, 4.75, 6.60 mg/g in 6 h, respectively, and the adsorption quantity of 60 nm PS-NPs is 0.43, 3.92, 4.39, 10.74, 20.20 mg/g in 6 h, respectively. In general, particle sizes have a great influence on the sorption behavior of MPs, and 500 nm PS-MPs and 60 nm PS-NPs have an obvious difference in the study. The results suggest that 60 nm PS-NPs have a stronger sorption of TC than 500 nm PS-MPs.

### 3.4. Cell Viability Assay

**Figure 4:**
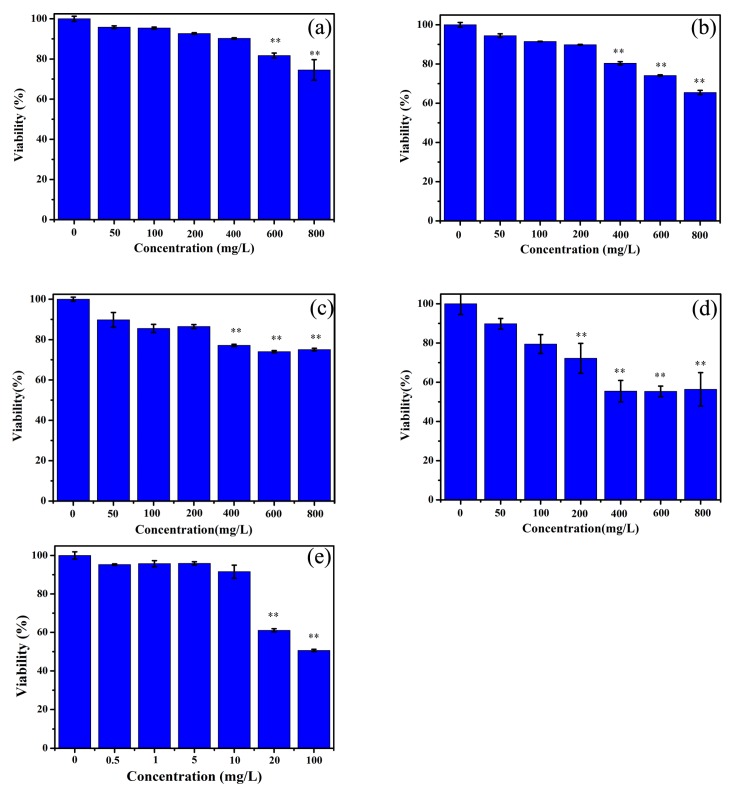
Cell viability assay. (**a**) Cell viability of exposing 500 nm PS-MPs (50, 100, 200, 400, 600, 800 mg/L); (**b**) Cell viability of exposing 500 nm PS-TC MPs (50, 100, 200, 400, 600, 800 mg/L); (**c**) Cell viability of exposing 60 nm PS-NPs (50, 100, 200, 400, 600, 800 mg/L); (**d**) Cell viability of exposing 60 nm PS-TC NPs (50, 100, 200, 400, 600, 800 mg/L); (**e**) Cell viability of exposing TC (0.5, 1, 5, 10, 20, 100 mg/L); (**: *p* < 0.01).

In Figure 4a, AGS cell viability decreases to 80 percent with 600 mg/L 500 nm PS-MPs exposure for 24 h. There is no obvious toxicity under 400 mg/L 500 nm PS exposure. As shown in Figure 4b, AGS cell viability decreases to 80 percent with an PS-TC NPs concentration between 400 mg/L and 600 mg/L. In Figure 4e, 20 mg/L TC there is clearly decreased cell viability. In Figure 4d, AGS cell viability has an apparently declining co-culture with 60 nm PS-NPs (400, 600, 800 mg/L) rather than 500 nm PS-MPs. What’s more, cell viability has no large diversity among 400, 600, 800 mg/L. PS-NPs. As shown in Figure 4f, 60 nm PS-TC NPs have induced the biggest lethal effect compared to 60 nm PS-NPs and 500 nm PS-TC NPs. These results indicate that a high concentration of PS has a low toxicity. However, the damage mechanism of PS-NPs and PS-MPs need to be analyzed from more dates.

### 3.5. Intracellular ROS

**Figure 5:**
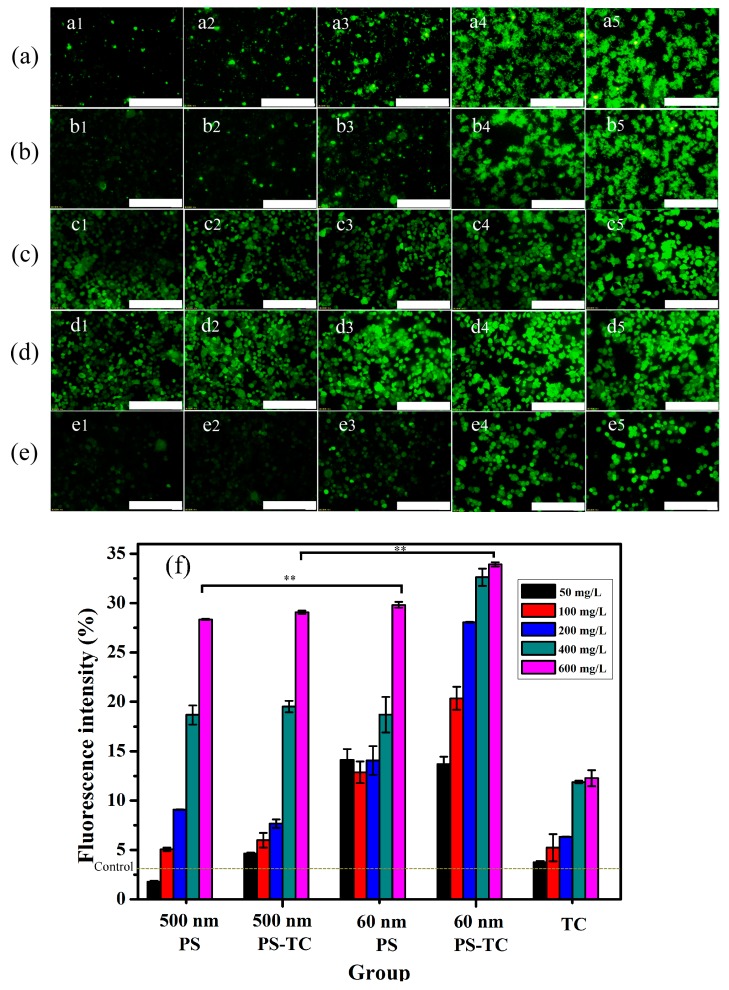
Fluorescence images and intensity of AGS cells. (**a**) Fluorescence images of exposing 500 nm PS-MPs (50, 100, 200, 400, 600 mg/L); (**b**) Fluorescence images of exposing 500 nm PS-TC MPs (50, 100, 200, 400, 600 mg/L); (**c**) Fluorescence images of exposing 60 nm PS-NPs (50, 100, 200, 400, 600 mg/L); (**d**) Fluorescence images of exposing 60 nm PS-TC NPs (50, 100, 200, 400, 600 mg/L); (**e**) Fluorescence images of exposing TC (0.5, 1, 5, 10, 20 mg/L); (**f**) Proportion of fluorescence intensity in Figure 5a–e; (scale: 250 μm; **: *p* < 0.01).

In this study, intracellular ROS levels are detected using an inverted Olympus fluorescence microscope. As shown in Figure 5, intracellular ROS levels are induced by PS and ROS levels have an increasing trend with increasing PS concentrations. It is obvious that oxidative stress has been triggered in Figure 5a,b, while AGS cells are exposed with 400 and 600 mg/L 500 nm PS-MPs and PS-TC MPs. What is more, in Figure 5c,d, 50, 100, 200, 400, 600 mg/L 60 nm PS-NPs and PS-TC NPs induce intracellular ROS apparently increasing compared with the control. As shown in Figure 5f, the fluorescence intensity of all exposure groups is analyzed using Image J. There is a significant difference between 500 nm PS-MPs and 60 nm PS-NPs in Figure 5f. The same result is determined between 500 nm PS-TC MPs and 60 nm PS-TC NPs. The results prove that 60 nm PS-NPs have a more significant effect on AGS cells than 500 nm PS-MPs.

### 3.6. Apoptosis Experiment

**Figure 6:**
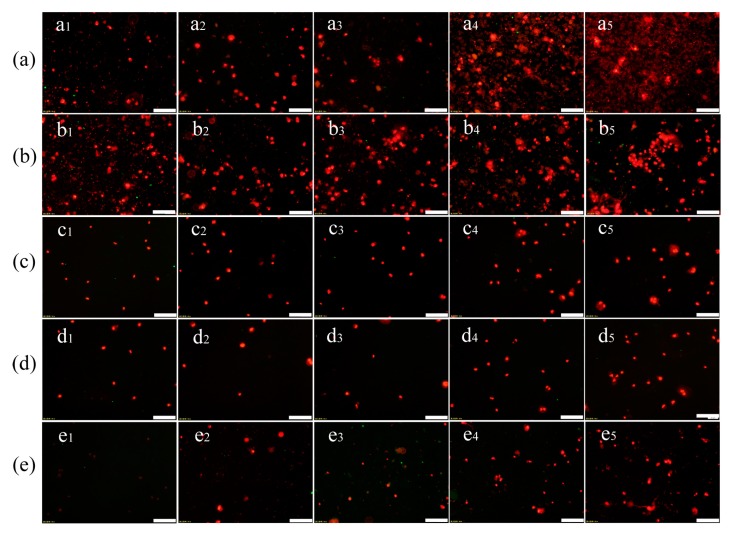
Fluorescence images of AGS cell apoptosis and fluorescence intensity. (**a**) Fluorescence images of exposing 500 nm PS-MPs (50, 100, 200, 400, 600 mg/L); (**b**) Fluorescence images of exposing 500 nm PS-TC MPs (50, 100, 200, 400, 600 mg/L); (**c**) Fluorescence images of exposing 60 nm PS-TC NPs (50, 100, 200, 400, 600 mg/L); (**d**) Fluorescence images of exposing 60 nm PS-NPs (50, 100, 200, 400, 600 mg/L); (**e**) Fluorescence images of exposing TC (0.5, 1, 5, 10, 20 mg/L); (scale: 250 μm).

In Figure 6, it is apparent that nuclei are stained with red. The result proves that PS-NPs and PS-MPs induce AGS apoptosis. Because there is no obviously green fluorescence in Figure 6, which is the symbol of early apoptosis, it can be concluded that PS-NPs and PS-MPs did not induce early apoptosis of AGS cells. As shown in Figure 6a–d, the number of AGS cell apoptosis significantly increases as PS-NPs and PS-MPs concentrations increase. In particular, 500 nm PS-TC MPs induce AGS apoptosis easier than 500 nm PS-MPs at the same concentration. As shown in Figure 6c,d, the results suggest that 60 nm PS-NPs and PS-TC NPs also induce AGS apoptosis, but there is little diversity between 60 nm PS-NPs and PS-TC NPs. In Figure 6e, 10 and 20 mg/L TC also induce AGS cell apoptosis. Therefore, it can be concluded that PS-MPs, PS-NPs, and TC in high concentrations can induce AGS cells apoptosis.

### 3.7. Comet Experiment Results

**Figure 7:**
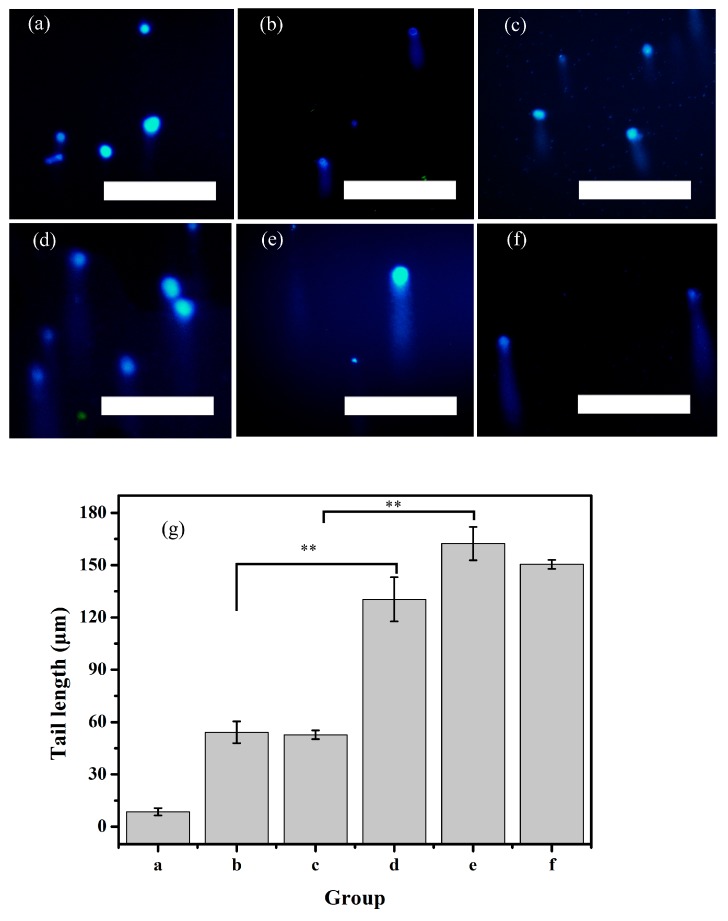
Comet assay. (**a**) Control; (**b**) 600 mg/L 500 nm PS-MPs; (**c**) 600 mg/L 500 nm PS-TC MPs; (**d**) 600 mg/L 60 nm PS-NPs; (**e**) 600 mg/L 60 nm PS-TC NPs; (**f**) 20 mg/L TC; (**g**) The tail length of Figure 7a–f; (scale: 250 μm; **: *p* < 0.01).

In Figure 7, there is an obvious tail among all exposure groups via the electrophoresis. Tail lengths of all exposure groups are longer compared with the control in Figure 7a. As shown in Figure 7b,c, the data demonstrates that both 600 mg/L 500 nm PS-MPs and PS-TC MPs have induced a certain amount of tail. Tail length has a little diversity between 500 nm PS-MPs and PS-TC MPs in Figure 7g. Both groups cause AGS cell DNA damaged in some way. Similarly, it is noteworthy that the cell tail is longer in Figure 7d,e. In detail, tail length of 600 mg/L 60 nm PS-TC NPs is three times that of the 500 nm PS-TC MPs exposure group in Figure 7g, and 60 nm PS-TC NPs induce significantly more damage to AGS than PS-NPs. It also determines that 20 mg/L TC also damaged AGS DNA seriously in Figure 7f. The results provide the evidence that PS-MPs can cause cellular damage at the genetic level. This damage could cause other physical or chemical injuries to AGS cells.

### 3.8. Apoptotic Protein Expression

**Figure 8:**
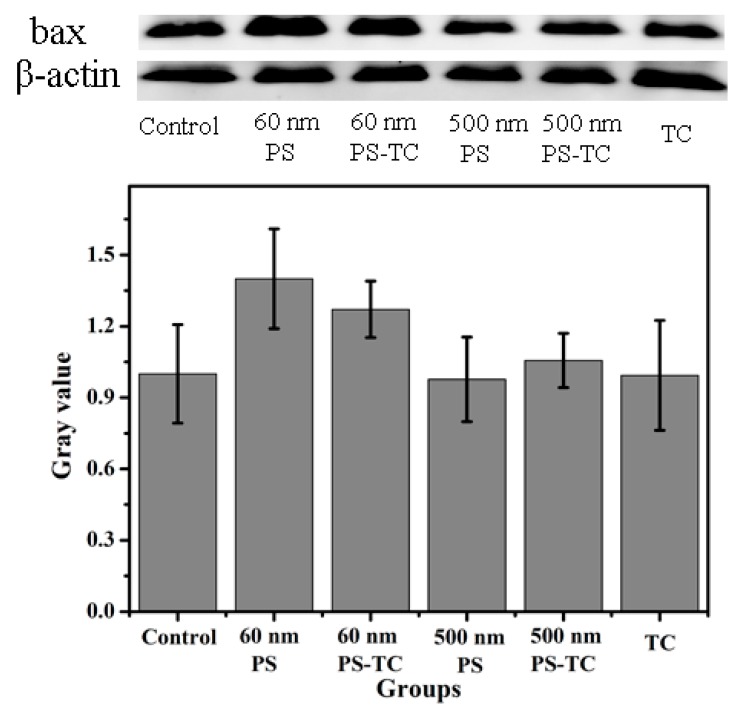
Apoptosis protein expression. The expression level of Bax protein in all groups (60 nm PS-NPs, 60 nm PS-TC NPs, 500 nm PS-MPs, 500 nm PS-TC MPs, TC).

In Figure 8, it is apparent that the gray values are higher in 60 nm PS-NPs and 60 nm PS-TC NPs than in the control. Both of them promote Bax overexpression and induce AGS apoptosis. However, 500 nm PS-MPs and 500 nm PS-TC MPs do not obviously promote apoptosis. The reason for this may be that NPs are smaller and enter AGS cells easily for 24 h acute exposure, meaning that 500 nm PS-MP may adsorb lots of protein in medium and their sizes are bigger than 500 nm. It is difficult to go through cell member. Therefore, PS-NPs promote AGS apoptotic easier than PS-MPs. This proves that NPs induce more damage to AGS than MPs.

## 4. Discussion

According to an investigation [37], PS particles have a considerably different adsorption to antibiotics because of their different functional groups. The adsorption capacity is positively related to TC concentrations. In the study, the hydration diameters of PS-NPs and PS-MPs in medium are larger in water because their surfaces absorb a level substance. We infer that PS-NPs’ and PS-MPs’ surfaces may combine with proteins or protein fragments in medium. Some researchers found that MPs can interact with digestive tract cells and NPs can go through the digestive tract [38,39,40]. The major adverse effects on organisms may be caused by MPs, NPs, and complex. Therefore, in order to study the adverse effects of 60 nm PS-NPs, 500 nm PS-MPs and PS-TC on humans, AGS cells are selected in this case. The research results suggest that 60 nm PS-NPs and 500 nm PS-MPs below 400 mg/L do not decrease cell viability obviously, which indicates a low cytotoxicity of PS-NPs and PS-MPs. Intracellular ROS levels and apoptosis are analyzed as two basic cytotoxicity functions. A study has confirmed that ROS levels might be different due to the various experimental conditions and species [41]. The results show that 200, 400, 600 mg/L 60 nm PS-NPs and PS-TC NPs inducing intracellular ROS increased significantly, and 400, 600 mg/L 500 nm PS-MPs and PS-TC MPs also induce and increase in intracellular ROS levels. Besides, the apoptotic results indicate that 600 mg/L PS-MPs, PS-TC MPs induce AGS apoptosis. Both 600 mg/L 60 nm PS-NPs and PS-TC NPs as well as 20 mg/L TC have some influence on AGS cell apoptosis. The results suggest that high concentrations of PS-NPs and PS-MPs can induce AGS apoptosis easier, but there is no obvious effect at a low concentration. It is reasonable to assume that protein coronas formed on the surface of PS-NPs and PS-MPs according to the previous study [42]. Protein coronas consist of biomolecules, proteins, and protein fragments. The forming of protein coronas may be due to chemical bonding and hydrogen bond interaction [42]. Their cytotoxicity may be weakened because protein coronas prevent PS-NPs and PS-MPs interacting with AGS directly.

Furthermore, both an endogenous and exogenous damage factor can induce DNA chain break. Comet results show that 600 mg/L 60 nm PS-NPs or PS-TC NPs damage DNA seriously compared with control, 600 mg/L 500 nm PS-MPs or PS-TC MPs. It proves that PS-NPs cause DNA damage more seriously than PS-MPs. The results seem to suggest that PS-NPs can go through cell membranes easily and cause genotoxicity. The above results provide insights into the different toxicities of 60 nm PS-NPs and 500 nm PS-MPs in AGS cells, and these data are very useful for human risk assessment.

## 5. Conclusions

In the study, adsorption experiment results demonstrate that 60 nm PS-NPs and 500 nm PS-MPs have an increased adsorption capacity to TC with the incensement of TC concentrations (1, 5, 10, 50, 100 mg/L) and that 60 nm PS-NPs and 500 nm PS-MPs can induce AGS cell damage. Furthermore, 600 mg/L PS-NPs and PS-MPs reduce cell viability, stimulate oxidation stress, and apoptosis. Sixty nanometer PS-NPs induce more serious damage than 500 nm PS-MPs in cell viability and intracellular ROS. DNA are also damaged by 60 nm PS-NPs and PS-TC NPs, 500 nm PS-MPs, and PS-TC MPs. What is more, 60 nm PS-NPs damages DNA more serious than 500 nm PS-MPs and 60 nm PS-NPs promote AGS apoptosis easier than 500 nm PS-MPs. All results show that oxidative stress and apoptosis-related signaling pathways may be activated. The study results provide evidence that PS-NPs and PS-MPs compound with TC, causing AGS cell damage under 24 h exposure.

## Figures and Tables

**Table 1:** The hydrated sizes of PS-NPs and PS-MPs.

Groups	Average Size (nm)
60 nm PS-NPs	155.64 ± 48.97
60 nm PS-NPs in medium	354.25 ± 151.93
500 nm PS-MPs	685.43 ± 35.71
500 nm PS-MPs in medium	873.02 ± 27.70
